# Occupational health disorders among physical education teachers compared to classroom and subject specialist teachers

**DOI:** 10.3389/fpubh.2024.1390424

**Published:** 2024-06-19

**Authors:** Neja Markelj, Marjeta Kovač, Bojan Leskošek, Gregor Jurak

**Affiliations:** Faculty of Sport, University of Ljubljana, Ljubljana, Slovenia

**Keywords:** occupational health problems, health disorders, teachers, physical education teachers, musculoskeletal disorders, hoarseness, hearing disorders

## Abstract

During the course of their work, teachers may be subjected to conditions that cause different health problems. This study examines occupational health disorders in a representative sample of 858 teachers (528 female; age 44.0 ± 9.67 years) divided into three groups of teachers with specific occupational requirements: specialist physical education teachers (specialist PETs), classroom teachers, and specialist teachers. The number of health disorders in the last 12 months was recorded using the *Chronic Health Disorders Questionnaire*. The differences between the different types of teachers, controlled for sex and age, were analyzed using binary logistic regression. The results showed that 89% of teachers experienced colds as the most frequently reported health problem, followed by 58% for lower back problems, 57% for headaches, 51% for hoarseness, and 43% for neck problems. A binary logistic regression showed that specialist PETs were the group with the highest health risk. They were about twice as likely to have musculoskeletal or hearing disorders than the other two groups of teachers. They were also significantly more likely to suffer from hoarseness. Understanding these different health challenges is critical to developing targeted interventions and robust support systems. These interventions should include initiatives aimed at raising awareness of health risk factors, implementing injury interventions and vocal cord hygiene programs, making ergonomic adjustments, and promoting awareness of self-care (both mental and physical). Given that the teaching profession is currently struggling with an aging workforce and a shortage of teachers, addressing these challenges is critical to the continued well-being of the teaching professionals.

## 1 Introduction

The teaching profession is associated with occupational stress resulting from the specific physiological, psychological, and physical demands of the profession (1–4). Teachers cite psychosomatic disorders and the symptoms of burnout syndrome as the main reason for the higher turnover rate and early retirement of teachers compared to other areas of the public service (3, 5). However, the occupational health risk of teachers often leads to voice disorders (6–11) and musculoskeletal disorders (lower and/or upper back pain; neck/shoulder pain; upper and lower arm pain; wrist, elbow, hip, knee, and ankle/feet pain; upper and lower leg pain) (11–13). Other disorders or diseases that teachers often struggle with are varicose veins, high blood pressure, autoimmune diseases, cardiovascular diseases, and sinusitis (12).

Gender, age, duration of employment, education level, weekly working hours, unfavorable posture, personality traits, and quality of life have been identified as associated risk factors for musculoskeletal disorders in several studies (11, 13, 14). A higher prevalence of musculoskeletal disorders was found in women, older teachers, teachers with longer teaching experience, teachers exposed to ergonomic risk factors (prolonged standing or sitting, working at a computer, and working with heavy loads), and teachers with an unhealthy personal lifestyle (smoking, drinking, too little exercise).

Physical education teachers (PETs) are exposed to similar psychological stressors as teachers of other subjects (subject specialist teachers) (15). However, compared to other teachers, they are also exposed to unique somatic demands of their work (16, 17). PETs engage in strenuous physical activity while teaching (e.g., participating in demonstrations, drills, and assisting students with exercises) while surrounded by high levels of noise (e.g., poor acoustics, shouting, and bouncing balls). Research shows that the increased physical strain puts significant stress on PETs’ lower limbs (18) and back (19), cardiovascular system (20), and respiratory system (19, 21). If PETs do not adapt their way of working to the age- and injury-related challenges, they may not be able to work until the official retirement age (17). In addition, the high noise exposure in the sports halls is a specific source of stress for this subgroup (15), which can have a significant impact on the prevalence of voice (e.g., hoarseness, increased effort to speak, chronic dryness or pain in the throat) and hearing problems (aphonia and dysphonia) compared to other teaching colleagues (16, 22, 23). The prevalence of voice and hearing problems is higher in female PETs (23, 24).

Given the increase in retirement age due to the aging of European society (25, 26) and the shortage of teachers (27–29), we conducted a cross-sectional study to gain more insight into the epidemiology of occupational health problems of different groups of teachers. Although previous studies have looked at the health problems of teachers in general, more detailed research that distinguishes PETs and other teachers remains relatively limited. This article attempted to address this gap by highlighting the challenges faced by PETs. Understanding these differences is crucial for developing targeted preventive measures and support systems to improve teacher well-being. Therefore, the aim of our study was to determine the current prevalence of musculoskeletal injuries, other health issues, and voice and hearing disorders in three groups of teachers: specialists PETs, classroom teachers teaching PE in lower grades in Slovenia, and subject specialist teachers. We hypothesized that the clinical incidence of musculoskeletal injuries and voice and hearing disorders would be higher in specialist PETs compared to classroom and subject specialist teachers. Further research objectives were to investigate the association between specific risk factors (age, sex) and the incidence of health problems studied using an odds ratio analysis.

## 2 Materials and methods

### 2.1 Subjects

The study was conducted on a nationally representative Slovenian sample of 858 teachers (528 female; age 44.0 ± 9.67 years). To control for between-school variance in occupational health conditions (e.g., gym acoustics, classroom heating), 170 primary (students aged 6–14 years) and upper secondary schools (students aged 15–18 years) were randomly selected from the Slovenian school register (30, 31). A total of 28% of the schools were included in the sample. All PETs from the selected schools were invited to participate. The response rate was around 60%. In the second step, a matched-pair sampling method was used. For those who chose to participate, pairs of teachers from the same school were identified and invited to the study. Where possible, a classroom teacher and a subject specialist teacher from the primary school surveyed were selected for the specialist PET, both of whom were of a similar age and sex to the specialist PET. In a few cases (e.g., when it was not possible to find a subject with the same sex), only age was a criterion for selection. In the upper secondary schools, a subject teacher was selected who was similar to the specialist PET. Music teachers were excluded from the sample as they are generally more familiar with speech therapy. Thus, the sample comprised 340 specialist PETs (141 female; age 45.0 ± 9.04), 201 classroom teachers (184 female; 41.7 ± 7.75), and 317 subject specialist teachers teaching subjects other than PE (203 female; 42.7 ± 8.71).

### 2.2 Variables

We used a modified version of a previously conducted survey (18, 23). This survey consisted of the following parts: (1) demographic data (sex, age); (2) anthropometric data (height, weight, and waist circumference), (3) characteristics of the teachers’ workplace (length of work experience, weekly workload), (4) *International Physical Activity Questionnaire – Short Form* (32), and (5) *Chronic Health Disorders Questionnaire* (23).

For this article’s purposes, only the results on chronic health disorders are presented. They were examined using the *Chronic Health Disorders Questionnaire*. They were defined as overuse injuries and/or pain in specific joints (e.g., cervical spine pain, lower back pain) and disorders and problems related to the teacher’s profession (e.g., voice disorders, hearing disorders, colds, etc.) that recurred frequently and lasted longer than 1 year. Dependent variables were the number of each health disorder in the last 12 months.

### 2.3 Procedure

Once the principals had given their consent, specially trained university physical education (PE) students visited the schools, conducted interviews with the teachers, and recorded their responses on a web-based form. The teachers were informed of the aims of the study and that their participation was voluntary and anonymous. The Ethics Committee of the Faculty of Sport in Ljubljana approved the study.

### 2.4 Data analysis

The data was analyzed using IBM SPSS 27 software (33).

The sample statistics of the variable distributions were calculated and plotted. For this purpose, the number of health problems in the last 12 months was merged into three categories: never (health disorder never occurred in the last 12 months), rarely (health disorder occurred 1–10 times in the last 12 months), and often (health disorder occurred more than 10 times in the last 12 months).

The differences between the different teacher types, controlled for sex and age, were analyzed using binary logistic regression; for this analysis, the categories rarely and often were combined.

## 3 Results

The study participant characteristics are listed in Table 1. The average female teacher was 43 years old and had over 18 years of teaching experience, while the average male teacher was 44 years old and had over 17 years of teaching experience. Both women and men work approximately 20 h per week and three additional hours for other educational activities. The female and male specialist PETs were on average 1–4 years older than the teachers in the other two subgroups and therefore had up to 5 years more teaching experience, while the workload was similar.

**Table 1:** Descriptive statistics of teachers’ characteristics.

Characteristic	Teachers	Male			Female			Total		
		*N*	*XA*	*SD*	*N*	*XA*	*SD*	*N*	*XA*	*SD*
Age (years)	PE	199	44.8	9.7	141	45.4	8.1	340	45.0	9.0
	Classroom	17	40.6	7.9	184	41.8	7.8	201	41.7	7.8
	Specialist	114	43.1	9.8	203	42.4	8.0	317	42.7	8.7
	Total	330	44.0	9.7	528	43.0	8.1	858	43.4	8.7
Body mass index (kg/m^2^)	PE	199	25.4	2.6	140	22.9	3.1	339	24.3	3.1
	Classroom	17	25.7	3.6	182	23.3	3.3	199	23.5	3.4
	Specialist	114	25.8	3.4	200	23.2	4.1	314	24.1	4.1
	Total	330	25.5	2.9	522	23.1	3.6	852	24.1	3.5
Waist circumference (cm)	PE	189	88.4	9.2	134	77.3	9.7	323	83.8	10.9
	Classroom	16	89.3	11.5	171	78.9	9.5	187	79.8	10.1
	Specialist	112	91.8	13.2	194	78.2	10.4	306	83.2	13.2
	Total	317	89.6	11.0	499	78.2	9.9	816	82.6	11.7
Years of teaching (years)	PE	199	18.6	10.4	141	20.8	9.0	340	19.5	9.9
	Classroom	17	13.4	6.8	184	18.3	8.9	201	17.9	8.9
	Specialist	114	16.6	10.2	203	17.4	8.8	317	17.1	9.4
	Total	330	17.6	10.2	528	18.6	9.0	858	18.2	9.5
Teaching hours per week (hours)	PE	199	20.2	3.9	141	20.7	3.2	340	20.4	3.6
	Classroom	17	21.8	6.5	184	21.1	4.0	201	21.2	4.3
	Specialist	113	20.1	5.8	203	19.6	5.4	316	19.8	5.5
	Total	329	20.2	4.8	528	20.4	4.5	857	20.4	4.6
Other organized educational activities (hours/week)	PE	196	3.5	4.4	141	3.3	4.5	337	3.4	4.4
	Classroom	17	3.3	4.8	181	2.9	3.7	198	2.9	3.8
	Specialist	113	3.3	5.0	199	3.2	4.9	312	3.2	4.9
	Total	326	3.4	4.6	521	3.1	4.4	847	3.2	4.5

N, Number of teachers; XA, Average; SD, Standard deviation.

**Figure 1:**
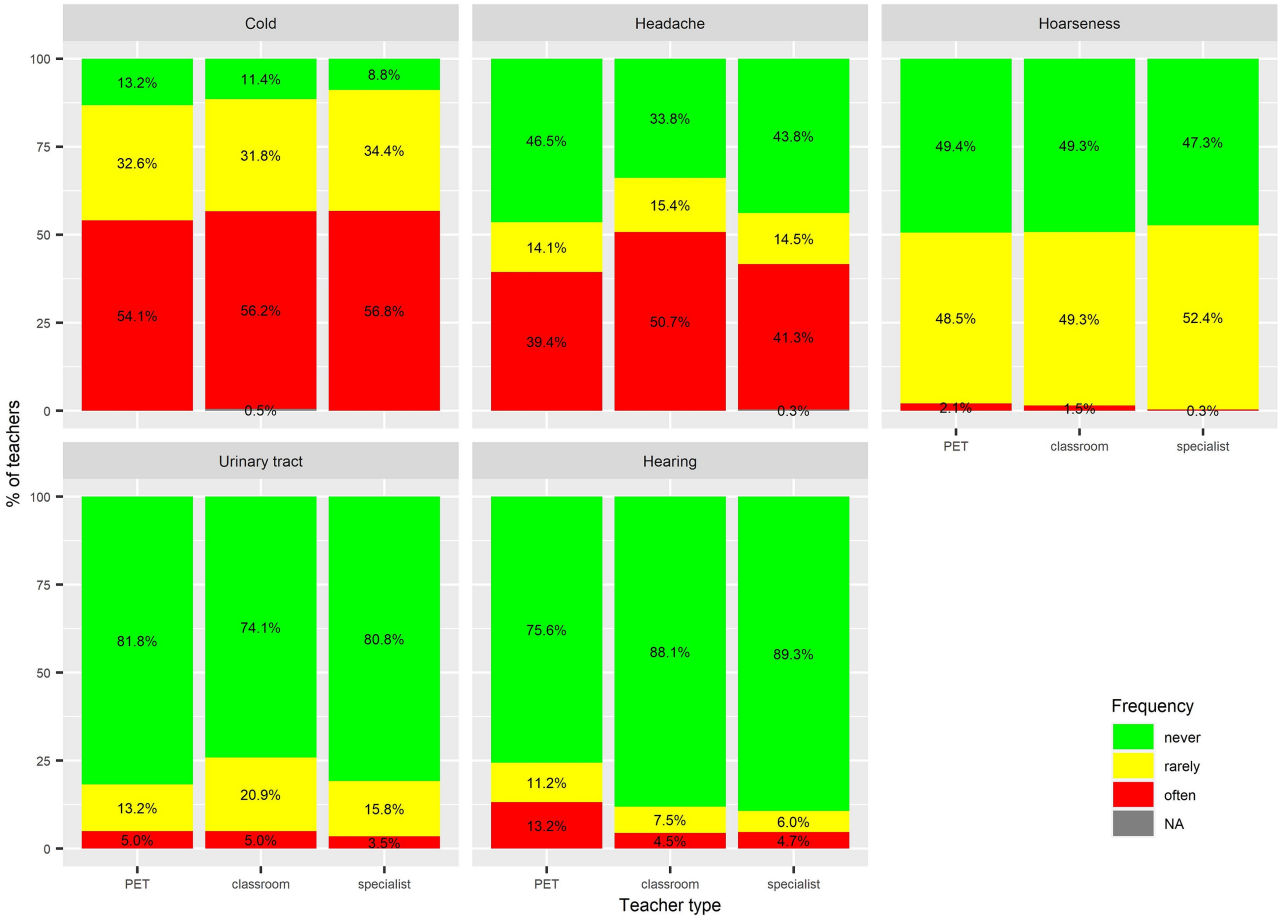
Basic statistics on frequent occupational health problems among teachers in the last 12 months.

**Figure 2:**
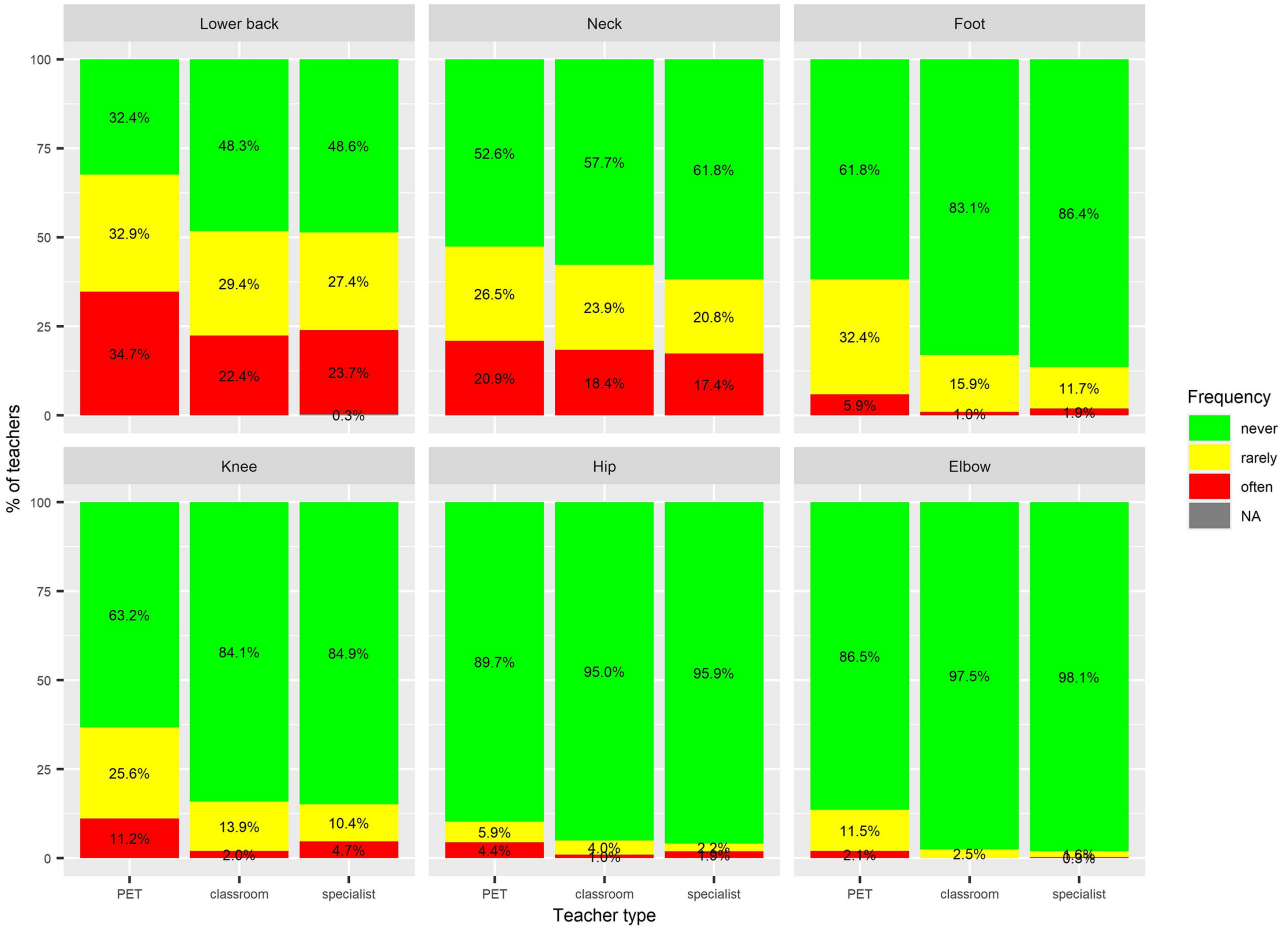
Basic statistics on musculoskeletal problems among teachers in the last 12 months. Values for *Hip problems* in specialist teachers: 2.2% rarely and 1.9% often. Values for *Elbow problems* in specialist teachers: 1.6% rarely and 0.3% often.

The results (Figures 1, 2) showed that colds were the most frequently reported health problem: 89% of respondents were affected by them and 56% stated that they occur frequently. Other common problems were lower back problems (58% affected; 28% frequently affected) headaches (57%; 43%), hoarseness (51%; 1%), and neck problems (43%; 19%). Less common health issues, each affecting less than a quarter of respondents, included problems with the feet, knees, urinary tract, hearing, hips, and elbows.

The frequency analysis indicated differences in musculoskeletal and hearing problems between specialist PETs and the other two groups of teachers. Binary logistic regression with controlled predictors for age and sex (Table 2) revealed that the adjusted odds ratios (*AOR*) for health problems among specialist PETs differed significantly from those of classroom and subject specialist teachers in several categories. The probability of having a headache was higher for classroom teacher (*AOR* = 1.4) when compared to specialist PETs. The probability of catching a cold was higher for subject specialist teachers when compared to specialist PETs (*AOR* = 1.51).

**Table 2:** Binary logistic regression adjusted odds (*AOR*) of the predictors for teachers’ health problems in the last 12 months.

	Predictor			
Health problem in last 12 months	Teacher type = classroom teacher	Teacher type = specialist teacher	Age (years)	Sex = female
Hoarseness	0.86^*^	1.01	1.00	1.34^*^
Cold	1.03	1.51^*^	1.01^*^	1.40^*^
Hearing	0.51^*^	0.42^***^	1.06^***^	0.96
Headache	1.40^*^	1.01	1.00	1.45^*^
Urinary tract	1.17	0.95	1.03^**^	2.39^***^
Neck	0.72^*^	0.66^*^	1.02^**^	1.47^*^
Lower back	0.66^*^	0.58^**^	1.06^***^	0.79^*^
Hip	0.55^*^	0.43^*^	1.10^***^	1.43^*^
Knee	0.47^**^	0.36^***^	1.05^***^	0.65^*^
Foot	0.38^***^	0.28^***^	1.03^**^	0.88^*^
Elbow	0.20^**^	0.14^***^	1.05^**^	0.96

Significance levels: ^*^5%, ^**^1%, ^***^0.1%. Reference categories are PETs and males.

As expected, the likelihood of health problems increased with age, except hoarseness and headaches. Sex differences were notable: women were significantly more likely than men to have a health problem, particularly urinary tract problems (*AOR* = 2.39), colds (*AOR* = 1.4), headaches (*AOR* = 1.45), neck problems (*AOR* = 1.47), and hip problems (*AOR* = 1.43). Conversely, women were significantly less likely to have lower back (*AOR* = 0.79), knee (*AOR* = 0.65), and foot problems (*AOR* = 0.88).

## 4 Discussion

This is one of the few studies that examined the occupational health problems of three groups of teachers depending on their specific working environment: specialist PETs, classroom teachers and subject specialist teachers. The main findings of this study were: (a) the most frequently reported health problems of teachers were colds, lower back problems, headaches, hoarseness, and neck problems; (b) among teachers, specialist PETs were the group with the highest health risk; (c) there were differences in teachers’ work-related health problems according to sex; and (d) health problems increased with the age of teachers.

### 4.1 Teachers’ general health

The results of this study are consistent with the existing literature on the prevalent health problems among teachers and point to occupational health risks [e.g., (8, 11, 16, 18, 23, 34–36)]. The increased incidence of colds and headaches among all teachers may be attributed to their increased susceptibility to viral infections due to regular contact with numerous children (37). In addition, somatic problems often associated with the stress of the teaching profession may contribute to these health problems as well (5).

The recurrence of lower back problems, hoarseness, and neck pain underpins a well-established trend in the teaching profession that has been observed in various studies [e.g., (7, 8, 11, 12)]. Prolonged standing and awkward posture may contribute to lower back pain (38), while neck pain may be associated with occupational stress (39). The prevalence of hoarseness is related to occupational demands for extensive verbal communication, which often takes place in noisy learning environments (6, 9, 40, 41). In particular, studies indicate a higher incidence of voice disorders in teachers compared to the general population, ranging from 32 to 58%, as opposed to 1% (6, 34, 42). These disorders are likely to be multifaceted and influenced by environmental, organizational, and predisposing factors that may exacerbate or trigger voice-related problems (43).

A comparative analysis of the health problems of different groups of teachers shows that the challenges for specialist PETs go beyond those of classroom and subject specialist teachers. They are about twice as likely to have musculoskeletal or hearing problems than the other two groups of teachers, and the risk of suffering from hoarseness is also significantly higher. These results are consistent with earlier findings (18, 19, 44).

### 4.2 Musculoskeletal disorders in teachers

The reported prevalence of musculoskeletal disorders in PETs varies widely in the literature, particularly for back pain, ranging from 4.7 to 76.7% (35). In our study, one-third of specialist PETs reported having had no problems with back pain in the last 12 months, while another third suffered from frequent back pain. The prevalence of neck pain reported in the literature is around 10% (35), while a higher prevalence (20.9% often and 26.5% rarely) was found among specialist PETs in our study. In addition, Slovenian specialist PETs reported knee (25.6% rarely and 11.2% often) and hip (5.9% rarely and 4.4% often) problems more frequently than their Estonian male counterparts (14% knee pain and 3.9% hip pain) (45).

The higher incidence of musculoskeletal health problems in specialist PETs compared to other teachers may be interpreted as a cumulative effect of workload on their musculoskeletal system (46), with activities such as demonstrations, overuse, collisions and carrying objects identified as major causes of injuries in PE (16). A systematic review by Erick and Smith (14) found a positive association between working more than 35 h per week, supervising students in a stooped posture and lifting sports equipment with low back pain. Intense physical activity during leisure time was also positively correlated with neck and knee pain.

Although Slovenian specialist PETs seem to have better working conditions than their international counterparts (e.g., most gyms have wooden floors and working hours are well below 35 h per week), the reasons for the higher prevalence of musculoskeletal disorders among them should be further investigated.

### 4.3 Hearing and voice disorders in teachers

A significant group of common occupational health problems faced by PETs concerns hearing (16, 23, 47) and voice disorders (8, 23, 24, 36). In a meta-analysis by Cantor Cutiva et al. (8), the prevalence of voice problems in teachers ranged from 9 to 37%, with a prevalence of 15–80% within 12 months. Allen and Hu (6) attribute the variability in prevalence to the different terms and definitions of voice disorders in the literature, ranging from general terms such as “voice complaints” to more specific definitions. In our study, about half of the teachers reported having suffered from voice disorders in the last 12 months. The probability of having voice disorders was higher for specialist PETs compared to classroom teachers and there was no difference when compared to subject specialist teachers. The literature suggests that working in noisy environment, as is characteristic of PE, and teaching younger students is associated with a higher incidence of voice disorders in teachers (8), which is not consistent with our findings. We would expect the likelihood of voice disorders to be higher among classroom teachers and specialist PETs than among subject specialist teachers. This discrepancy may suggest that classroom teachers compared to other teachers are more aware of the importance of voice care and may incorporate voice production techniques and voice care principles, such as appropriate hydration, into the classroom. Similar may also apply to PETs compared to other subject specialist teachers.

In addition, the prevalence of hearing disorders in specialist PETs is at least twice as high as in other teacher groups in Slovenia. About a quarter of Slovenian specialist PETs reported having hearing problems (often or rarely), in contrast to 65% of Brazilian teachers (25% with some degree of measured hearing loss) (48) and 46% of Swedish teachers (49).

Several factors contribute to these disorders in teachers. These include environmental, organizational, and predisposing elements that may exacerbate or trigger voice disorders (43). Key risk factors for voice disorders include high noise levels, poor acoustics, gender, upper respiratory problems, caffeine consumption, speaking loudly, teaching hours per week, and experience of dismissal due to voice problems (7, 50). In addition, PETs often work in an environment with dust and multiple classes practicing at the same time (16), which has a negative impact on teachers’ voices. Another important factor contributing to the development of voice disorders is also a lack of knowledge about voice production techniques and principles of voice care (6).

A higher risk of developing hearing disorders in PETs is also associated with several factors (23, 47, 51), including working in spaces with high reverberation, exposure to loud activities, dust and concurrent teaching, all of which are common in PE (16, 52). Noise exposure, particularly in indoor swimming pools and triple gyms, often exceeds acceptable levels for acoustic comfort, reaching around 80 dB(A) (53). Acceptable levels for acoustic comfort are up to 55 dB(A) (54). Sudden loud noises, such as children shouting, and consistent noise levels in classrooms can lead to lesions in the inner hair cells over time (55).

Given these conditions, the hearing health of specialist PETs is of particular concern. This reflects previous research findings that emphasize the increased risk of hearing damage in this subgroup (23, 47, 51). In addition, the fact that PETs are exposed to noise for prolonged periods of time without adequate protection, often in an attempt to discipline students or counteract environmental noise (e.g., bouncing balls or music), puts stress also on their vocal organs and contributes to vocal problems such as hoarseness, increased effort when speaking, chronic dryness, or pain in the throat (8, 16, 23, 24, 47). The prevalence of voice and hearing problems tends to be higher in female PETs (23, 24).

### 4.4 Differences in occupational health problems among teachers according to sex and age

Our study also shows some sex differences in occupational health problems among teachers. Female teachers have a higher risk of urinary tract problems, hoarseness, colds, headaches, and neck and hip problems than their male counterparts, which has already been confirmed in previous studies [e.g., (6, 35)]. A higher risk of voice disorders in female teachers has also been found in other studies [e.g., (40, 42, 44)]. This can be partly explained by gender-specific differences in the larynx (56). In addition, social and cultural factors (e.g., greater vocal demands in class to maintain class order) may contribute to speech problems in females (41). More frequent colds, headaches, and hip and neck problems in females than males are consistent with trends in the general population (57–60). In contrast, the male teachers in our study were more prone to musculoskeletal problems, with the exception of hip problems. This could be explained by their recreational sports activities, where males choose activities with a higher risk of musculoskeletal problems compared to females (e.g., ball and racket games) (61) or they practice moderate and vigorous intensity sports more often than females (62).

In line with the literature [e.g., (3, 11, 18)], the results of this study also emphasize the positive association between teacher’ occupational health problems and age, with the exception of hoarseness and headaches. We can assume that, as in the general population, teachers’ health problems are influenced by aging processes (63, 64) as well as by the cumulative effect of workload in a given work environment (i.e., vocal demands, many encounters with people, rigorous schedule, standing for long hours) (6, 65, 66).

### 4.5 Possible prevention measures

Understanding the specific health challenges faced by different groups of teachers is therefore crucial for developing tailored interventions and support systems to prevent their work-related health problems (22). Interventions may include raising teachers’ awareness of health risk factors, teaching appropriate tasks to reduce specific health risks (e.g., voice care or injury prevention), introducing school-based exercise for them to increase their general health and well-being, ensuring outdoor teaching conditions, ergonomic adaptations in the classroom (e.g., lifting tables that allow sitting or standing) and, specifically for PETs, teaching strategies for managing physical exertion that meet the particular demands of PE.

Injury prevention measures have already proven successful in reducing injuries among PETs. Vercruysse et al. (67) familiarized an intervention group of PETs with eight injury prevention strategies (seven intrinsic strategies: correct execution, warm-up, cool-down, stretching, core stability, balance, and functional strength and one extrinsic: appropriate footwear) in a 2-day training session. The results showed that teachers in the intervention group had a lower number of injuries per 1,000 h of instruction than the control group and used a broader range of injury prevention strategies. Similar results can also be seen in vocal hygiene awareness programs for teachers. Allen and Hu (6) reported that teachers who had participated in such a program (tips on vocal use and misuse, the importance of drinking water, taking vocal breaks, and avoiding caffeine) showed a statistically significant increase in their awareness of vocal care 3 months later.

As the teaching profession struggles with an aging workforce (25, 26) and a teacher shortage (27–29), addressing these challenges is critical to maintaining a healthy teaching environment that is attractive for future teachers and for teachers to stay in the teaching profession.

### 4.6 Study strengths, limitations, and future directions

The strength of the study lies in the matched-pair sampling method, which controls for hidden variables and eliminates the order effect. The sample is therefore representative nationwide.

Although this study provides valuable insights, certain limitations must be acknowledged. The cross-sectional design limits causal inferences, and future longitudinal studies could examine the trajectory of health problems among teachers. In addition, consideration of general lifestyle factors and psychological well-being could improve the comprehensive understanding of occupational health in the teaching profession.

### 4.7 Conclusion

Our study contributes to a more nuanced understanding of teachers’ occupational health problems and provides insights into the challenges specialist PETs face compared to their teaching colleagues, such as constant physical exertion in a noisy environment. These challenges, if not addressed, could lead to long-term health consequences. This reflects previous studies that have pointed to the significant physical strain of PETs, particularly on the musculoskeletal system and hearing. These findings serve as a basis for training future teachers and for targeted interventions to improve teachers’ overall wellbeing, ultimately promoting a healthier and more sustainable teaching workforce.

## Data availability statement

The raw data supporting the conclusions of this article will be made available by the authors, without undue reservation.

## Ethics statement

The studies involving humans were approved by Ethics Committee Faculty of Sport, University of Ljubljana. The studies were conducted in accordance with the local legislation and institutional requirements. Written informed consent for participation was not required from the participants or the participants’ legal guardians/next of kin because it was not required by Ethics Committee.

## Author contributions

NM: Writing – original draft, Writing – review & editing. MK: Conceptualization, Investigation, Supervision, Writing – review & editing. BL: Data curation, Formal analysis, Methodology, Writing – review & editing. GJ: Conceptualization, Funding acquisition, Project administration, Supervision, Writing – review & editing.
